# Changes in soil bacterial community characteristics in patches of different vegetation types under different stages of restoration in the desert of northern China

**DOI:** 10.1002/ece3.11353

**Published:** 2024-07-23

**Authors:** Haonian Li, Yumei Liang, Zhongju Meng, Xiaomen Ren, Ruibing Meng, Feiyan Zhao

**Affiliations:** ^1^ College of Desert Control Science and Engineering Inner Mongolia Agricultural University Hohhot China; ^2^ Inner Mongolia Institute of Meteorological Sciences Hohhot China

**Keywords:** Hobq Desert, plant patch type, recovery phase in sandy lands, soil bacterial diversity and community composition, soil quality

## Abstract

In desert areas, the process of mobile sandy land changing to semi‐fixed sandy land and eventually to fixed sandy land after undergoing vegetation restoration is inevitable. The presence of shrub patches and herb patches is common in this restoration process. No relevant studies have reported the soil bacterial community characteristics of different vegetation‐type patches (shrub patches and herb patches) under different stages of restoration. Therefore, we utilized long‐established experimental plots to collect soil from 0–20 cm soil layer under shrub patches (dominated by *Salix psammophila*) and herb patches under different stages of restoration (i.e., mobile sand land, semi‐fixed sand land, and fixed sand land), by determining soil physicochemical properties, enzyme activities, and soil bacterial communities. Our results found that soil bacterial α‐diversity under different restoration stages showed higher shrub patches than herb patches. The dominant bacterial communities (phyla) in shrub patches and herb patches at different recovery stages were *Actinobacteria*, *Proteobacteria*, and *Bacteroidota*. When the mobile sandy land returned to fixed sandy land, the relative abundance of *Actinobacteria* and *Bacteroidota* gradually decreased under shrub patches and herb patches, while the relative abundance of *Proteobacteria* increased significantly. In addition, herb patches significantly increased the relative abundance of bacteria (genus) relative to shrub patches at different stages of recovery. Soil nutrients, soil fine particles, and soil enzyme activities were significantly higher under shrub patches than under herb patches when fixed sandy land due to differences in life form and architecture between shrub patches and herb patches. Based on this, soil bacterial community composition and diversity under shrub patches were driven by more soil properties during the restoration of sandy land. This study complements the dynamic recovery processes and driving mechanisms of soil bacterial community structure under different vegetation patches in sandy areas, especially in the context of global climate change.

## Graphical abstract

In desert areas, the process of mobile sandy land changing to semi‐fixed sandy land and eventually to fixed sandy land after undergoing vegetation restoration is inevitable. The presence of shrub patches and herb patches is common in this restoration process. No relevant studies have reported the soil bacterial community characteristics of different vegetation‐type patches (i.e., shrub patches and herb patches) under these three restoration stages (i.e., mobile sandy, semi‐fixed sandy, and fixed sandy).

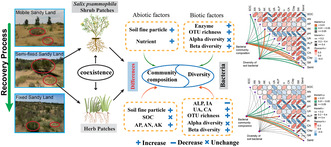

## 1 INTRODUCTION

The transformation of global climate patterns and the effects of overgrazing have made desertification one of the major global ecological problems affecting human survival and sustainable development (Huang et al., 2020). The process of desertification has spread to about 40% of the global land area, spanning multiple climatic zones and ultimately leading to a sustained loss of life‐sustaining ecosystem services (D'Odorico et al., 2013). Countries around the world have carried out relative desertification control projects. Africa has built a “Great Green Wall” in the Sahel region to promote the improvement of human health and well‐being (Mirzabaev et al., 2021). Spain mitigates regional soil erosion through the implementation of forestry programs (Romero‐Diaz et al., 2010). The Chinese government has also implemented ecological restoration programs such as the Three‐North Shelter Forest Program (Zhai et al., 2023). In the process of natural recovery, the age of plant cover and biomass increase, and the habitat for survival forms a unique pattern of resource‐limited distribution (i.e., mobile sandy land gradually evolves into semi‐fixed sandy land and eventually into fixed sandy land) (Liu et al., 2016). Previous studies have mainly confirmed that the species diversity of aboveground vegetation communities increases significantly from mobile sandy land to fixed sandy land and that soil nutrients and enzyme activities depend on the increase in plant diversity (Haotian et al., 2019; Zhang et al., 2023). In contrast, more research needs to be done on soil bacterial communities during this process. As one of the wealthiest and most diverse microorganisms, soil bacteria are essential in maintaining soil structure, nutrient transformation (Lopez‐Lozano et al., 2016), ecosystem stability, and regulating plant growth and interspecific plant competition (Po‐Ju Ke, 2019). However, the biotic and abiotic factors that drive the characterization of soil bacterial communities are not consistent, as they are influenced by the degree of ecological restoration of the region. For example, a meta‐analysis identified soil organic carbon and N content as drivers of enrichment of bacterial community diversity (Huang et al., 2022). At the same time, Liang et al. (2021) reported that changes in soil bacterial community composition under different recovery stages of plantation forests promoted the accumulation of available phosphorus. In contrast, scientists agree that the pH is the best indicator for driving soil bacterial community structure (Liu et al., 2023). In addition, soil enzyme activities drive nutrient management and metabolic activities of soil microbial communities (Huang et al., 2022). In a previous study, Huang et al. (2022) reported that planting vegetation significantly increased soil urease, alkaline phosphatase, and catalase activities relative to degraded land. Soil enzyme activity is affected by factors such as the stage of vegetation restoration and vegetation type. As the time of vegetation restoration increased, significantly increased urease, alkaline phosphatase, dehydrogenase, catalase activities (Ren et al., 2016). Zhang et al. (2012) observed that soil oxidases recovered faster than hydrolase activity as vegetation restoration proceeded in the desert, with oxidase activity showing a peak recovery rate during the shrub‐dominated area, whereas the recovery of hydrolase activity appeared to be regulated primarily by herb. It can be seen that differences in vegetation patches and degree of restoration highly significantly affect the utilization of soil enzyme activities. People gradually realize the significant influence of environmental restoration on the interaction between soil and microorganisms (Chen et al., 2021). However, the main functional of regulating soils in desert regions differ from those in other regions due to the arid and high‐temperature climatic conditions, and the specific climatic conditions alter the adaptive strategies of bacterial communities and the utilization of enzyme activities (Piotrowska‐Długosz et al., 2022; Tóth et al., 2017). Therefore, we must still add information about soil bacterial communities in desert ecosystems during different restoration processes.

The coexistence of shrub patches and herb patches is a common feature in desert areas (Biancari et al., 2023). Once shrub seedlings are established, they can better trap wind‐borne sediments, concentrating under the shrub canopy and decreasing between shrubs (Pierce et al., 2019). This process also promotes the recovery of herbaceous species. There are fewer studies on the shrub patches and herb patches that form after development from mobile sandy land to fixed sandy land. More studies have focused on changes in soil bacterial communities under different stages of restoration of the same vegetation type, while neglecting the contribution of herb, which is widely distributed between shrub and shrub, to soil properties. For example, Liang et al. (2021) reported significant differences in soil bacterial community diversity and structure under *Pinus massoniana* in different stages of restoration, with the dominant microbial community shifting from oligotrophic to eutrophic. In addition, a number of scholars have assessed the recovery of vegetation and soil properties in mobile sandy land, semi‐fixed sandy land, and fixed sandy land. Liu, Li, et al. .(2021) confirmed that soil bacterial characteristics were significantly correlated with plant abundance, cover, and biomass. In particular, plant species were important determinants in building bacterial composition. Zuo et al. (2016) further confirmed that soil properties, plant biomass, and the development of soil microbial communities are closely related. It can be seen that the impact of differences in vegetation patch types on the characteristics of soil bacterial communities from mobile sandy land to fixed sandy land still needs to be clarified. Fine delineation of different vegetation patches will help improve our understanding of the dynamics of below‐ground bacterial communities at different stages of restoration. This will improve our ability to protect and restore desert ecosystems.

Here, we conduct relevant experiments within the long‐term experimental observation area. The dominant shrub species in the experimental observation area is *Salix psammophila*, as it plays an essential ecological role in desert restoration and management in northern China. We selected *Salix psammophila* as a study object because it plays an important ecological role in the restoration and management of desert ecosystems (Zhao et al., 2021). In addition, we selected herb patches between shrub patches, and the herb patches were mainly dominated by *Agriophyllum squarrosum*, *Corispermum hyssopifolium*, and *Psammochloa villosa*. We determined soil physicochemical properties, enzyme activities, and bacterial community characteristics of shrub patches and herb patches in the desert under different stages of restoration. Our objective was to assess soil bacterial communities in shrub patches and herb patches under different stages of restoration. We hypothesized that shrub patches and herb patches would exhibit different response strategies in bacterial community composition under different recovery stages. In addition, as the recovery stage progresses, differences in the recovery rates of soil physicochemical properties and enzyme activities under shrub patches and herb patches result in different factors driving bacterial community structure. Based on these hypotheses, we want to elucidate the effects of shrub patches and herb patches on soil bacterial community characteristics at different stages of desert restoration; and assess the drivers of soil bacterial community characteristics under herb and shrub patches.

## 2 MATERIALS AND METHODS

### 2.1 Investigation of study area

**FIGURE 1:**
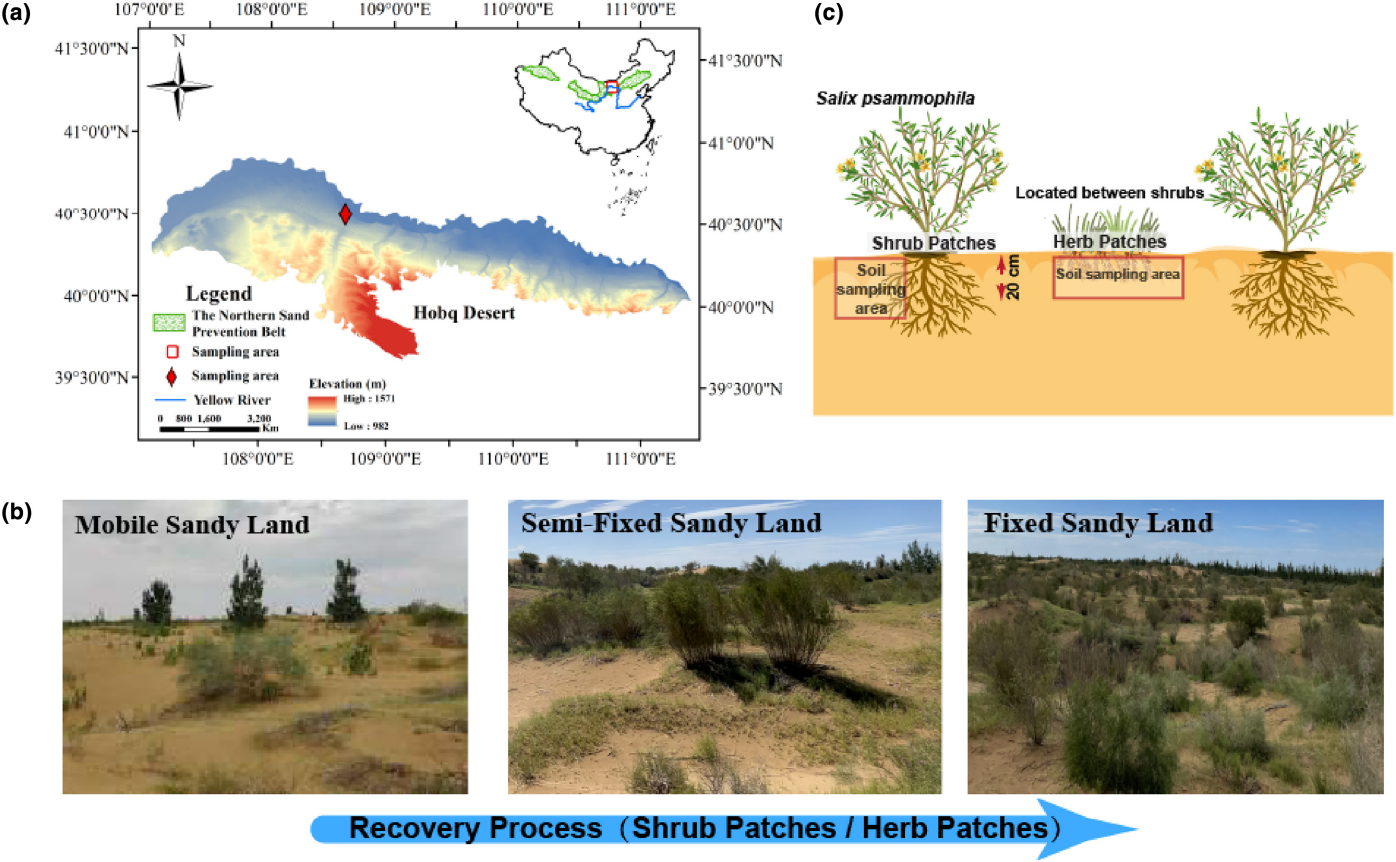
Location of the Hobq Desert study area (a). Mobile (Mobile Sandy Land), Semi‐Fixed (Semi‐Fixed Sandy Land), Fixed (Fixed Sandy Land) (b). Schematic diagram of soil sample collection (c). This abbreviation also applies to other figures and tables.

This study is located in Hobq desert – Yellow River coastal ecotone (39°21′20″–40°51′49′ N, 106°54′15′′–109°15′10″ E) (Figure 1). The environment of this ecotone is fragile and prone to degradation due to strong climate change and anthropogenic disturbances (pollution and production construction) (Yu & Wang, 2018). The average annual precipitation is 188.4 mm. Active wind erosion tends to occur in spring and winter. The original landscape of the region is dominated by mobile sandy land, and previous studies have estimated the emissions of saltation in the region to be 31 t/m for the past two decades (Du, Xue, Deng, et al., 2015; Du, Xue, Wang, & Deng, 2015). In order to reduce the damage caused by wind and sand to the city and the Yellow River basin, forestry activities are carried out in the region. Plant species suitable for desertification control in northern China, such as *Hedysarum scoparium* and *Salix psammophila*, are mainly planted here.

### 2.2 Collection of soil sample

In this study, our experiment was conducted at the National Positional Observation and Research Station for Desert Ecosystems, Hangjinqi, Inner Mongolia. Changes in vegetation cover determine the restoration stage of the desert. Mobile sandy land is in the early stage of vegetation restoration, and the vegetation coverage is less than 15%. Semi‐fixed sandy land is in the middle stage of vegetation restoration, and the vegetation coverage is 15%–40%, fixed sandy land is in the late stage of vegetation restoration, and the vegetation coverage is more than 40% (Liu, Li, et al., 2021) (Figure 1). *Salix psammophila* is the dominant shrub in the study area and is approximately 15–20 years old in the test area (Figure 1). Considering the huge impact of soil moisture on vegetation growth in the experimental area, there is no irrigation activity during the growth period of *S. psammophila*. Under different stages of restoration, several herbaceous species common to the desert grew between shrubs, forming shrub patches and herb patches (Figure 1). Herb patches were mainly dominated by *Agriophyllum squarrosum*, *Corispermum hyssopifolium*, and *Psammochloa villosa* (Table 1). We set up three 30 m × 30 m experimental plots on mobile sandy land in the study area. In each experimental plot, we randomly selected five shrub patches (under the shrub canopy) and five herb patches (between shrub and shrub) for soil collection, and then minimized experimental error by mixing soil samples from the same plant patch type into one. The above soil collection steps were repeated three times. Since soil properties in the topsoil layer are widely affected by vegetation restoration, the 0–20 centimeters soil is the focus of this study (Gao et al., 2014) (Figure 1c). Soil samples were collected from 0–20 centimeters and were sieved through a 2 mm sieve to remove plant roots. The soil samples collected here are not rhizosphere soil, but bulk soil. After that, the same treatment is carried out under different stages of recovery. We collected a total of 18 soil samples. Each soil sample was divided into two subsamples, one subsample was stored in sterile centrifuge tubes to determine biological parameters, and the other subsample was brought back to the laboratory and air‐dried to determine physicochemical parameters.

**TABLE 1:** Primary conditions of different vegetation‐type patches at different stages of restoration.

Types		Main plant species	Above ground biomass
Mobile	Shrub Patches	*Salix psammophila*	6.17 ± 0.69 kg/plant
	Herb Patches	*Agriophyllum squarrosum, Corispermum hyssopifolium*	13.72 ± 0.43 g/m^2^
Semi‐fixed	Shrub Patches	*Salix psammophila*	13.80 ± 1.25 kg/plant
	Herb Patches	*A. squarrosum, C. hyssopifolium, P. villosa*	24.00 ± 9.99 g/m^2^
Fixed	Shrub Patches	*Salix psammophila*	12.11 ± 0.93 kg/plant
	Herb Patches	*C. hyssopifolium*	24.14 ± 2.63 g/m^2^

### 2.3 Determination of soil properties

Hydrochloric acid and hydrogen peroxide were used to remove salts and calcium carbonate from the soil samples, and soil particle size was determined using a FRITSCH particle size meter (Idar Oberstein, Germany). Following the soil particle grading standards developed by the U.S. Department of Agriculture (USDA), we categorize soil particle size into clay (0–0.002 mm), silt (0.002–0.05 mm), and sand (0.05–2 mm). The instrument is based on the laser scattering analysis method. The repeatability of the measurement results is ±0.5%, which complies with the international ISO13320 standard. Soil organic carbon (SOC) represents the carbon cycle and is the primary indicator of the unstable carbon sources available. Available potassium (AK), available phosphorus (AP), and alkaline nitrogen (AN) were selected as proxies for the K‐cycle, N‐cycle, and P‐cycle, representing the forms of K, N, and P available to plants and microorganisms (Li et al., 2021). The SOC was determined by the potassium dichromate‐external heating method (Li, Meng, et al., 2022). The AP was determined by 0.5 mol/L NaHCO_3_ method. AK was determined by the NH_4_Oac leaching‐flame photometric method (Li, Meng, et al., 2022). The AN was determined by hydrolytic diffusion of alkaline solution (Wu et al., 2020). The pH was determined by a 1:5 ratio of distilled water to the test sample.

The assay method of our chosen soil enzyme activity was carried out as described in the reports of Zhang et al. (2015) and Liu et al. (2014), using a colorimetric method. Catalase activity was determined by the decomposition of hydrogen peroxide. Urease activity was determined by the indophenol blue colorimetric method, which measures the water‐soluble dye indophenol blue at 578 nm. Invertase activity was determined by the 3,5‐dinitrosalicylic acid method. Alkaline phosphatase activity is measured colorimetrically by adding a substrate (p‐nitrophenyl phosphate) to the resulting product before analysis. DNA was extracted from the soil bacterial community using a DNA extraction kit (MagPure Soil DNA LQ Kit, D6356‐02), followed by agarose gel electrophoresis and NanoDrop2000 to detect the concentration of sample DNA. Genomic DNA was used as a template for PCR amplification using specific primers containing barcode and Tks Gflex DNA Polymerase (Takara, R060B) according to the selection of sequencing regions to ensure amplification efficiency and accuracy. We used primers 343F (5′‐TACGGRAGGCAGCAG‐3′) and 798R (5′‐AGGGTATCTAATCCT‐3′) to amplify the V3‐V4 variable region of the bacterial 16SrDNA gene (Nossa et al., 2010). PCR products are detected by electrophoresis and purified by magnetic beads. The amplification system was 30 μL. The mixture was 15 μL of 2 × Gflex PCR Buffer, 1 μL of 5 pmol/μL primer F, 1 μL of 5 pmol/μL primer R, ≥1 μL of Template DNA, 0.6 μL of Tks Gflex DNA Polymerase (1.25 U/μL), and make up to 20 μL with H_2_O. Reaction parameters: pre‐denaturation at 94°C for 5 min for 1 cycle, 26 cycles (denaturation at 94°C for 30 s, annealing at 56°C for 30 s, extension at 72°C for 30 s). The reaction parameters were 94°C pre‐denaturation for 5 min for 1 cycle, 26 cycles (94°C denaturation for 30 s, 56°C annealing for 30 s, 72°C extension for 20 s), and finally 72°C extension for 5 min. After purification, PCR products are quantified by Qubit. PCR products were aliquoted according to PCR product concentration and sequenced.

The bioinformatics analysis procedure uses cutadapt software to remove the primer sequences from the raw data sequence. The qualified paired‐ended reads from the previous step were then analyzed for quality control using DADA2 according to the QIIME 2 default parameters (Callahan et al., 2016). The ASV was tabulated using QIIME2, and all sequences were aligned to the Silva (version 13.8) database (Bolyen et al., 2019). Species comparison annotations were analyzed using q2‐feature‐classifier software default parameters (Chao & Bunge, 2002; Daly et al., 2018). We will use the cloud platform to analyze the α‐diversity Index (https://cloud.oebiotech.cn/task/). In addition, only species whose relative abundance of bacterial communities at the phylum level are within the top 10 and species whose relative abundance of bacterial communities at the genus level are within the top 20 in all soil samples are discussed in this study. The bacterial community at the phylum level with a relative abundance greater than 10% is regarded as the dominant bacterial community.

### 2.4 Statistical analysis of data

One‐way ANOVA was conducted using IBM SPSS 22.0 software (Chicago, USA), and a Duncan multiple comparison probability level of 0.05 was used to compare and contrast to determine significant differences in abiotic and biotic properties of the soil under different stages of restoration between shrub patches and herb patches. Significant differences in soil properties between herb and shrub patches were analyzed using independent samples t‐tests with probability levels of 0.05 and 0.01. The effects of different restoration stages, vegetation patch types, and different restoration stages × vegetation patch types on soil bacterial community composition were examined using principal component analysis (PCoA) and multivariate analysis of variance (PERMANOVAs) based on Bray‐Curtis distance. Mantel tests of soil properties were performed using R software version 4.2.0 (Auckland, New Zealand). Graphing was performed using Origin Lab software (Northampton, USA). The RDA (Redundancy analysis) analysis was used to detect soil environmental factors that influence the composition of soil bacterial communities. Linear discriminant analysis (LDA Effect Size) was used to evaluate high‐dimensional bacterial taxa and determine the enrichment of bacterial taxa.

## 3 RESULTS

### 3.1 Distribution of soil physical and chemical properties at different recovery stages

#### 3.1.1 Soil particle distribution under different recovery stages

**FIGURE 2:**
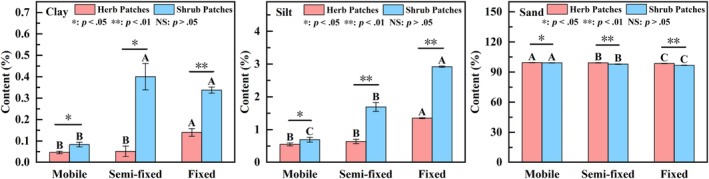
Soil particle distribution of herb patches and shrub patches under different recovery stages. Different capital letters indicate significant differences between recovery stages under the same vegetation patch type (*p* < .05). *, **, and NS represent significant relationships (*p* < .05), highly significant relationships (*p* < .01), and non‐significant relationships (*p* > .05) in soil properties between shrub patches and herb patches, respectively.

Soil particles in shrub patches and herb patches under different restoration stages were dominated by sand (values ranging from 96.74% to 99.40%) (Figure 2). Shrub patches and herb patches under different recovery stages significantly enhanced the distribution of the clay and silt particles and significantly reduced the distribution of sand particles to the soil (*p* < .05). Specifically, from mobile to fixed, soil clay and silt particles increased by 325% and 323% in shrub patches, respectively, and by 180% and 145% in herb patches, respectively. At different recovery stages, the clay and silt particles exhibited greater shrub patches than herb patches. In addition, there were apparent differences in clay, silt, and sand in the shrub and herb patches under different recovery stages, and these differences became more significant with the development of the recovery stages. In summary, shrub patches and herb patches effectively increased the accumulation of soil clay and silt particles with the development of recovery. The clay and silt were significantly higher under shrub patches than herb patches, and the difference between shrub patches and herb patches became stronger as recovery progressed.

#### 3.1.2 Distribution patterns of soil chemical properties under different recovery stages

**FIGURE 3:**
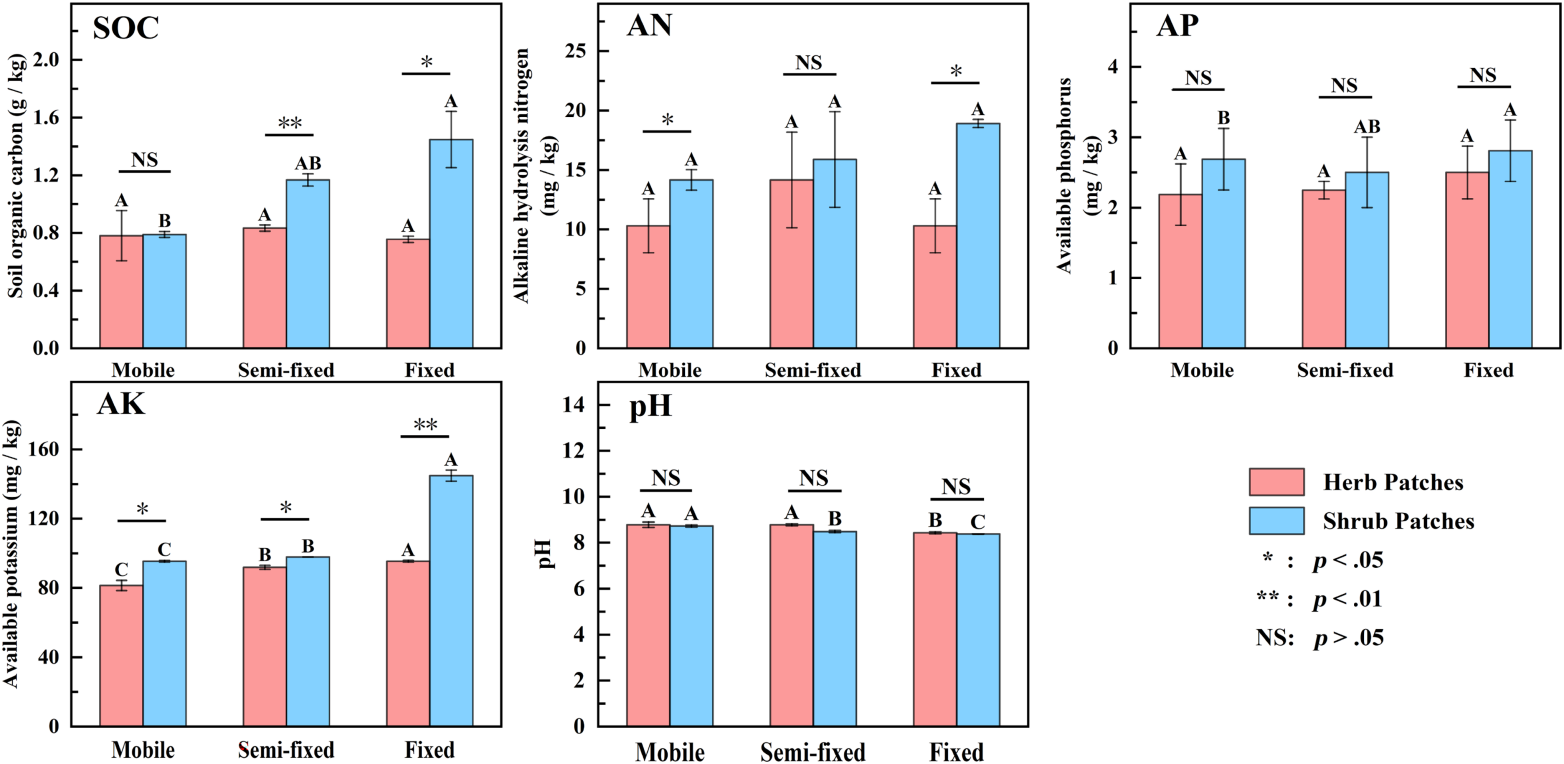
Soil particle distribution of herb and shrub plots under different recovery stages. Different capital letters indicate significant differences between recovery stages under the same vegetation patch type (*p* < .05). *, **, and NS represent significant relationships (*p* < .05), highly significant relationships (*p* < .01), and non‐significant relationships (*p* > .05) in soil properties between shrub patches and herb patches, respectively. AK, Available potassium; AN, Alkaline hydrolysis nitrogen; AP, Available phosphorus; SOC, Organic carbon.

From the mobile to fixed, shrub patches and herb patches promote soil nutrient accumulation (Figure 3). Except for the AN, the improvement of the SOC, the AP, and the AK by shrub patches differed significantly under different recovery stages. Compared with the mobile sandy land, the content of SOC, AP, and AN of the herb patches in the fixed sandy land increased by 28%, 64%, and 77%, respectively. The pH values of shrub patches and herb patches at different recovery stages ranged from 8.38 to 8.76 and 8.39 to 8.89, respectively. The soil in this study area was alkaline. The colonization of shrubs and herbs contributed to the decrease in pH value. In addition to the AP and pH, there were varying degrees of variability in SOC, AN, and AK between the shrub and herb plots, from mobile to fixed roughly showed that shrub patches were significantly higher than herb patches. In summary, soil nutrient accumulation favors pH reduction. There were significant differences in soil nutrients under shrub patches and herb patches with different recovery stage.

### 3.2 Distribution of enzyme activities under different recovery stages

**FIGURE 4:**
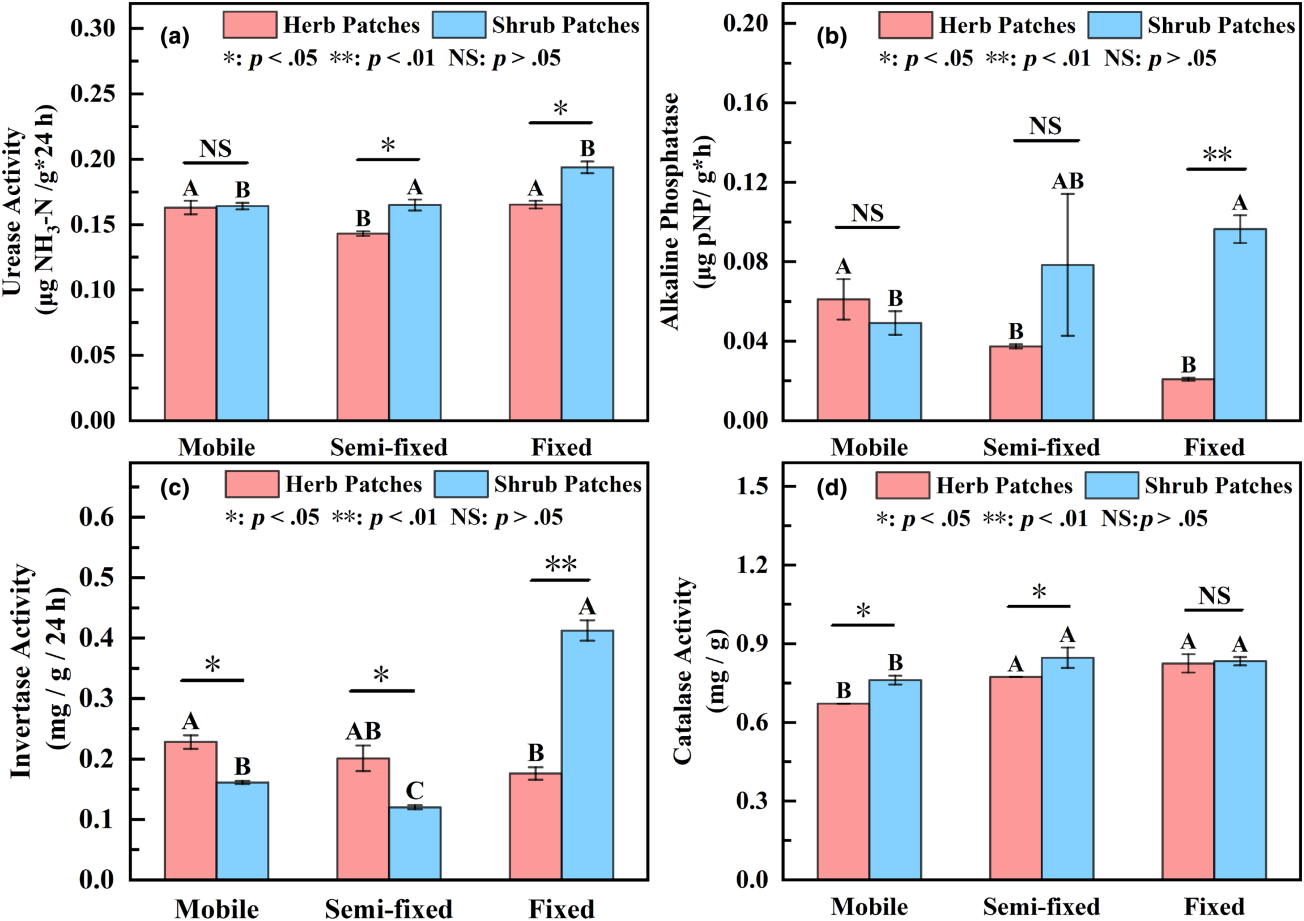
Distribution of enzyme activity of herb and shrub patches at different recovery stages. Different capital letters indicate significant differences between recovery stages under the same vegetation patch type (*p* < .05). *, **, and NS represent significant relationships (*p* < .05), highly significant relationships (*p* < .01), and non‐significant relationships (*p* > .05) in soil properties between shrubs and herbs, respectively. (a), (b), (c), and (d) represent the UA (urease), ALP (alkaline phosphatase), IA (invertase), and CA (catalase), respectively.

There were significant differences in the UA, ALP, IA, and CA at different stages of recovery (*p* < .05) (Figure 4). The soil enzyme activity under shrub patches increased significantly from mobile to fixed (*p* < .05), and except for the ALP and IA, the UA and CA significantly increased with the recovery stage under the herb patches (*p* > .05). Significant differences in IA were found in herb and shrub patches at different recovery stages (*p* < .05). From the mobile to fixed, the UA and ALP under shrub patches and herb patches changed from no significant difference to a significant difference. Thus, shrub patches and herb patches promoted soil enzyme activity under restoration. The effect of vegetation patch type on soil enzyme activity was influenced by the stage of restoration.

### 3.3 Soil bacterial community composition and diversity distribution under different recovery stages

**FIGURE 5:**
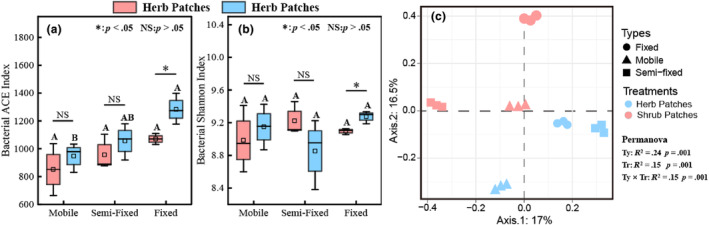
α‐diversity (a, b) and β‐diversity (c) of soil bacteria in herb and shrub patches at different recovery stages. Different capital letters indicate significant differences between recovery stages under the herb patches or shrub patches (*p* < .05). *, **, and NS represent significant relationships (*p* < .05), highly significant relationships (*p* < .01), and non‐significant relationships (*p* > .05) in soil properties between shrubs and herbs, respectively. (a), (b), and (c) represent ACE index, Shannon index, and PCoA analysis, respectively.

We chose the Shannon index and ACE index to indicate the α‐diversity of soil bacteria (Figure 5a,b). The Shannon index was insignificant in different recovery stages (*p* > .05). Except for the fixed, there were no significant differences in the Shannon index and ACE index in shrub and herb patches under different stages. Therefore, the recovery of soil bacterial communities under different recovery stages is slow. When reaching the fixed, there were significant differences in the diversity and number of species composition of soil bacterial communities under shrub and herb plots (i.e., shrub patches were larger than herb patches). Principal component analysis (PCoA) based on Bary‐Curtis distance was used to analyze the β‐diversity at different recovery stages and vegetation patch types (Figure 5c). The first and second axes explained 33.5% of the total variation of β‐diversity. Multiple permutation analysis (Permanova) demonstrated the β‐diversity for recovery stage, vegetation type, and recovery stage × vegetation type. Permanova revealed that the recovery stage, vegetation patch type, and recovery stage × vegetation patch type significantly affected the β‐diversity (*p* < .001).

**FIGURE 6:**
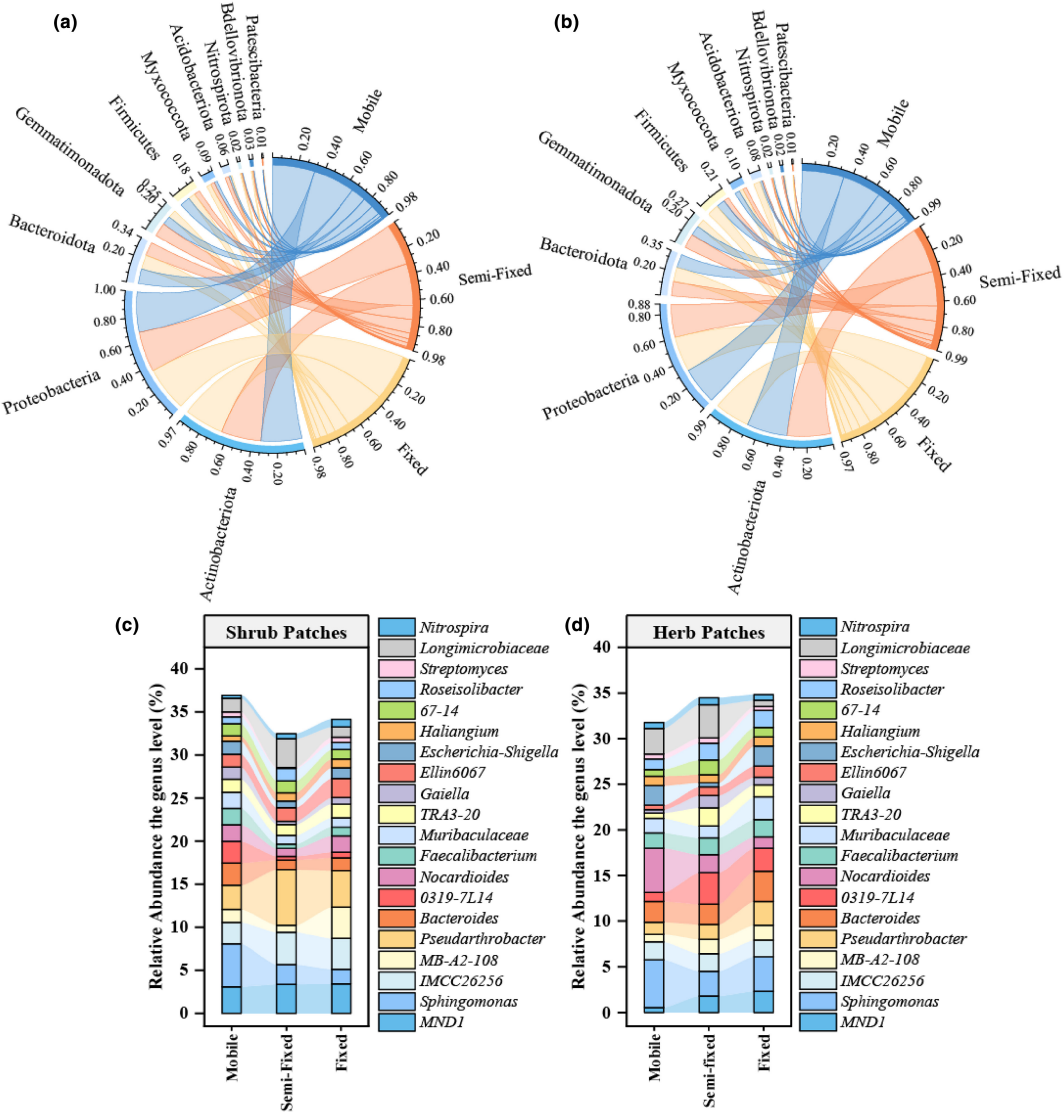
Relative abundance at the bacterial phylum (a, b) and genus (c, d) level of herb (b, d) and shrub patches (a, c) at different recovery stages.

**FIGURE 7:**
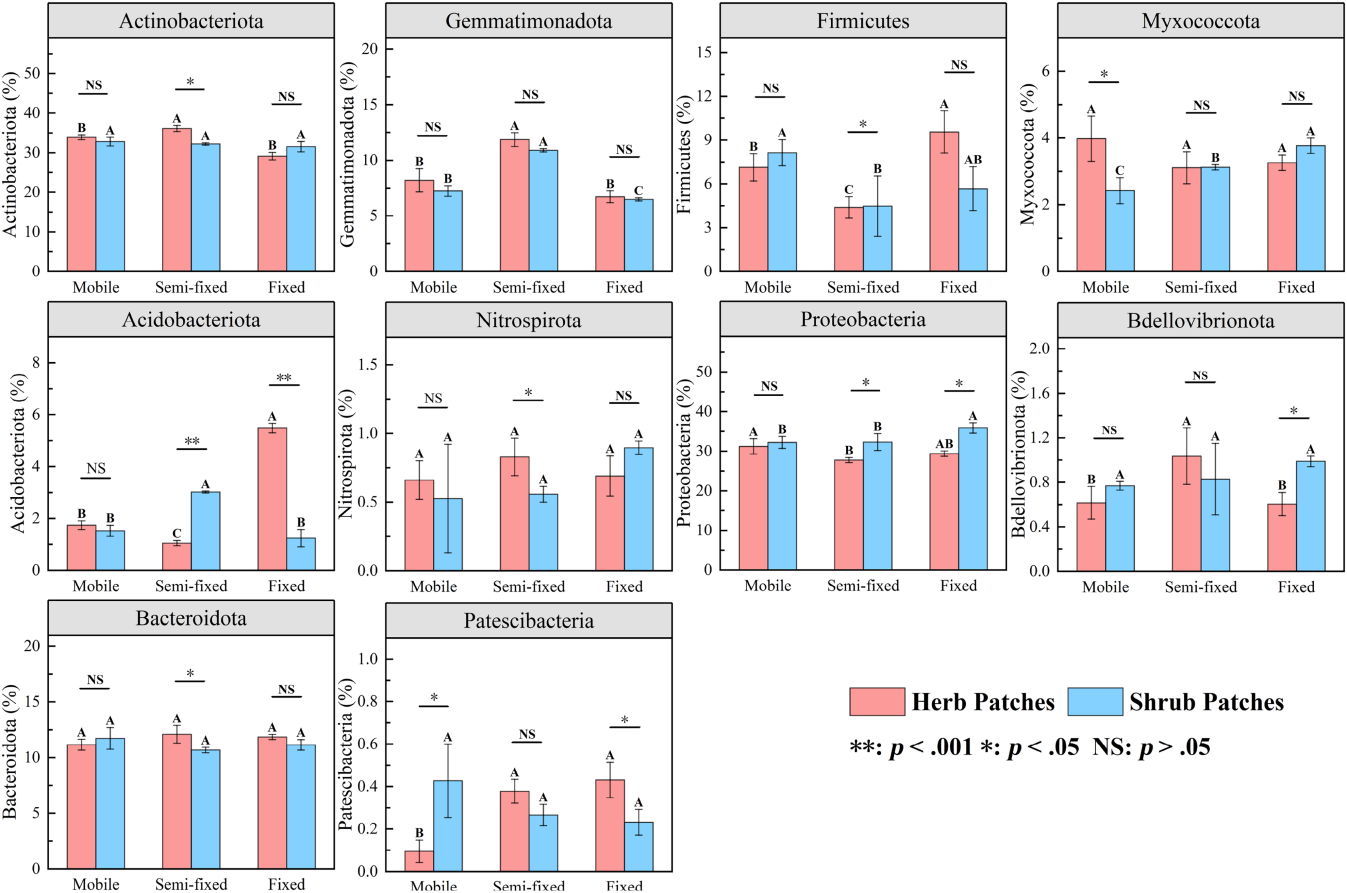
Relative abundance at the top 10 bacterial phylum level of herb and shrub patches at different recovery stages. Different capital letters indicate significant differences between recovery stages under the herb plots or shrub patches (*p* < .05). *, **, and NS represent significant relationships (*p* < .05), highly significant relationships (*p* < .01), and non‐significant relationships (*p* > .05) in soil properties between shrub patches and herb patches, respectively.

**FIGURE 8:**
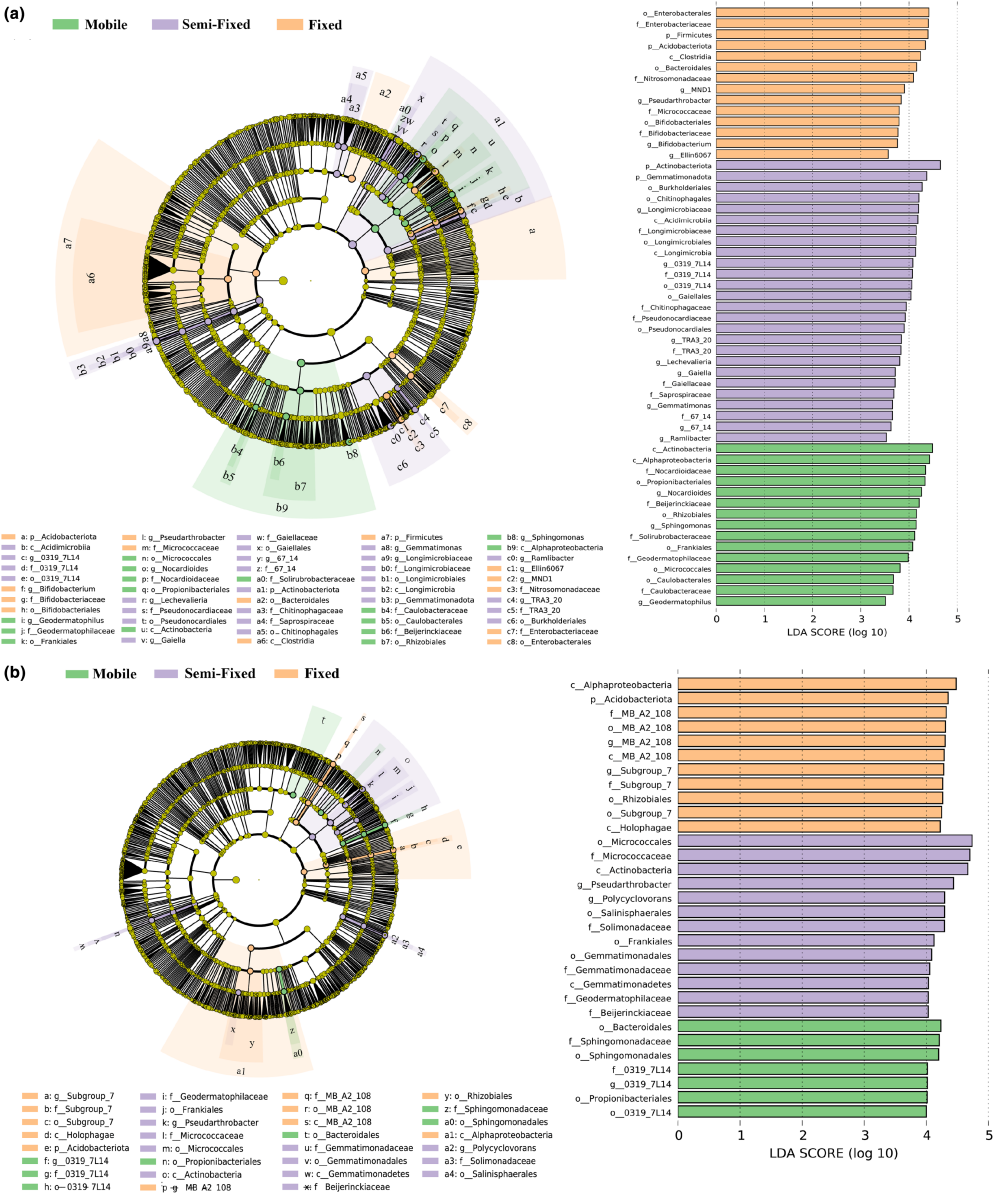
Cladogram phylogenetic maps of bacterial lineages in shrub (b) and herb (a) patches at different recovery stages. Taxa with significant differences in abundance are indicated by colored dots, and branch diagram circles indicate phylogenetic taxa from phylum to genus. Calculate of histograms of LDA scores for different abundant species in the bacterial communities under shrub and herb patches.

The top 10 bacterial phylum in the soil samples were *Actinobacteriota*, *Gemmatimonadota*, *Firmicutes*, *Myxococcota*, *Acidobacteriota*, *Nitrospirota*, *Proteobacteria*, *Bdellovibrionota*, *Bacteroidota*, and *Patescibacteria*. Except for *Nitrospirota*, *Bacteroidota*, and *Patescibacteria*, the rest of the phyla showed significant differences across recovery stages (*p* < .05) (Figure 6a,b). Specifically, *Bacteroidota*, *Proteobacteria*, and *Nitrospirota* showed a gradual increase in relative abundance from mobile to fixed. The relative abundances of *Actinobacteriota*, *Gemmatimonadota*, *Acidobacteriota*, *Bdellovibrionota*, and *Patescibacteria* showed a general increasing and decreasing pattern. Except for *Gemmatimonadota*, the remaining bacterial phyla differed significantly between shrub and herb patches under different stages (*p* < .05) (Figure 7). The different patch types of plants resulted in differences in the relative abundance of soil bacterial phyla. Recovery stages change the distribution pattern of the relative abundance of soil bacterial phyla. At the genus level, the top 5 genera for herb patches under mobile were *Sphingomonas*, *Nocardioides*, *Bacteroides*, *Longimicrobiaceae*, *Escherichia*‐Shigella, the top 5 genera for herb patches under semi‐fixed were *Sphingomonas*, *Bacteroides*, *0319‐7L14*, *Longimicrobiaceae*, *TRA3‐20*, the top 5 genera for herb patches under fixed were *Sphingomonas*, *Bacteroides*, *Pseudarthrobacter*, *0319‐7L14*, *Muribaculaceae* (Figure 6d). The top 5 genera for shrub patches under mobile were *Sphingomonas*, *MND1*, *Pseudarthrobacter*, *Bacteroides*, *0319‐7L14*, the top 5 genera for shrub patches under semi‐fixed were *MND1*, *IMCC26256*, *Pseudarthrobacter*, *Longimicrobiaceae*, *Sphingomonas*, the top 5 genera for shrub patches under fixed were *MND1*, *IMCC26256*, *MB‐A2‐108*, *Ellin6067*, *Pseudarthrobacter* (Figure 6c). According to LEfse analysis, there was a significant difference in bacterial community composition under shrub patches and herb patches (Figure 8). Fifty‐six and thirty‐one bacterial clades were statistically significantly different at the recovery level in shrub patches and herb patches, respectively. In the herb patches, in the mobile, *Alphaproteobacteria* (order), *Actinobacteria* (order), *Geodermatophilus* (from family to genus), *Nocardioidaceae* (from family to genus), and *Propionibacteriales* were heavily enriched (with a higher LDA SCORE); In the semi‐fixed, *Actinobacteriota* (phylum), *Gemmatimonadota* (phylum), *Burkholderiales* (order), *Longimicrobia* (from order to genus), and *0319_7L14* (from order to genus) were heavily enriched; in the fixed, *Enterobacterales* (from order to family), *Acidobacteriota* (phylum), *Firmicutes* (phylum), and *Clostridia* (order) were heavily enriched. In the shrub plots, *Bacteroidales* (order), *Sphingomonadaceae* (order to family), *0319_7L14* (order to genus), and *Propionibacteriales* (order) were heavily enriched in the mobile; *Micrococcales* (from order to family), *Gemmatimonadota* (from order to family), *Actinobacteriota* (order), *Pseudarthrobacter* (genus), and *Polycyclovorans* (genus) were heavily enriched in the semi‐fixed; *Alphaproteobacteria* (phylum), *Acidobacteriota* (phylum), *MB_A2_108* (from phylum to genus), *Subgroup_7* (from order to genus), *Rhizobiales* (order) were heavily enriched in the fixed. Overall, shrub patches possessed a higher relative abundance of genus bacteria than herb patches. Shrub and herb patches led to differences in the relative abundance of soil bacterial communities. Recovery stages change the distribution pattern of relative abundance of phylum and genus bacteria. The LEfes analyses revealed variability in the composition of bacterial communities at different recovery stages. In addition, the bacterial community composition of shrub patches and herb patches showed inconsistent patterns.

### 3.4 Relationship between soil properties and soil bacterial community characteristics

**FIGURE 9:**
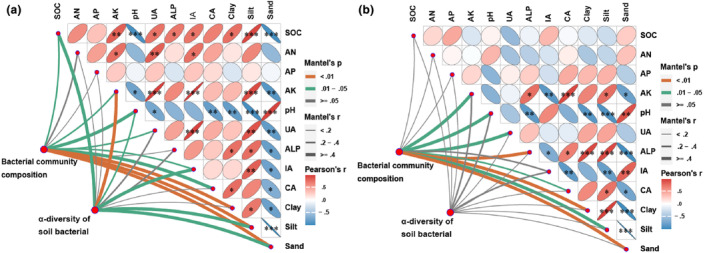
Relationship between soil bacterial community characteristics and soil properties in shrub patches (a) and herb patches (b) were examined by Mantel test. AK, Available potassium; ALP, Alkaline phosphatase; AN, Alkaline hydrolysis nitrogen; AP, Available phosphorus; CA, Catalase; IA, Invertase; SOC, Organic carbon; UA, Urease.

**FIGURE 10:**
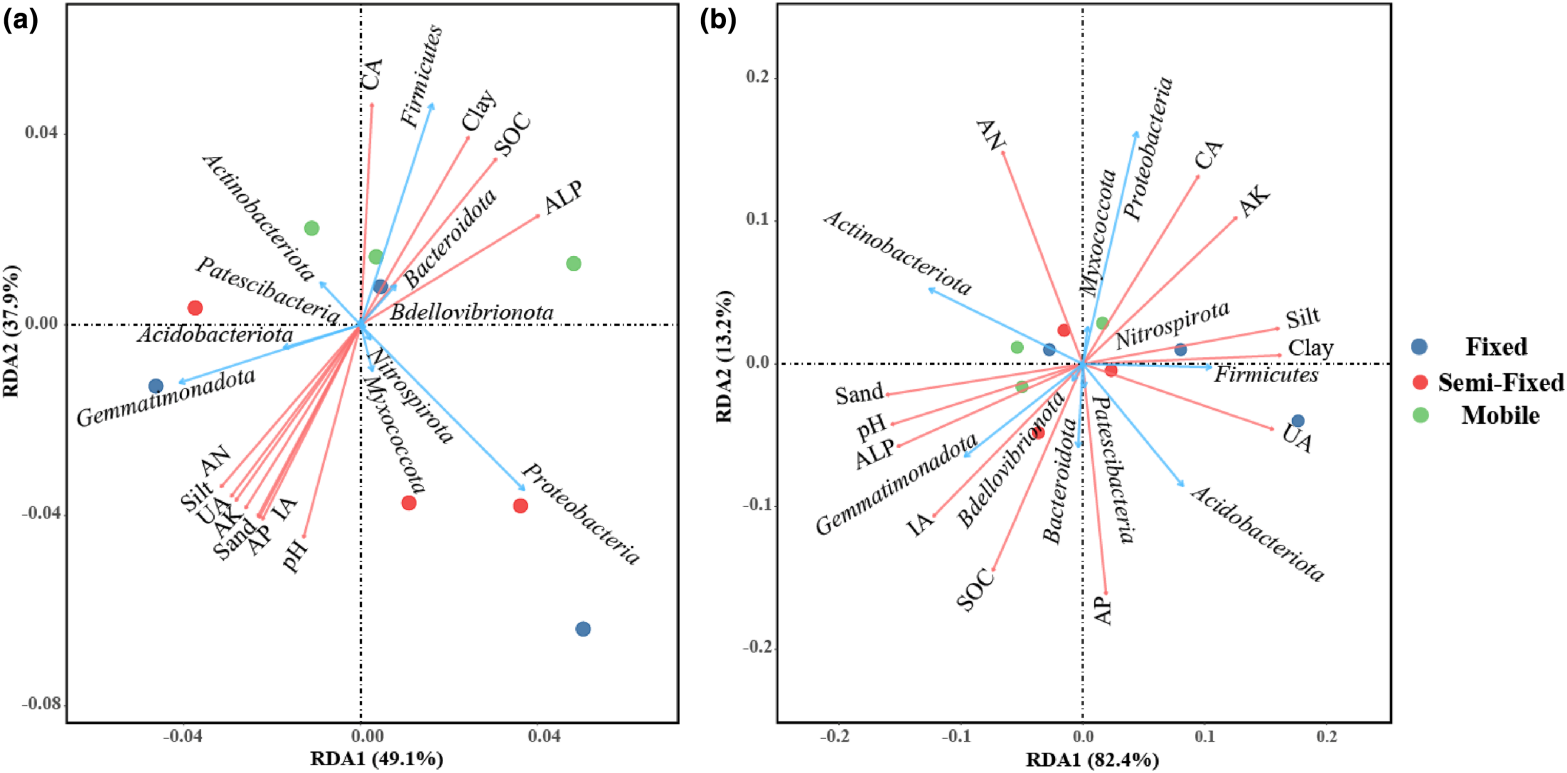
Correlation between bacterial phylum and soil properties of herb (b) and shrub patches (a). AK, Available potassium; ALP, Alkaline phosphatase; AN, Alkaline hydrolysis nitrogen; AP, Available phosphorus; CA, Catalase; IA, Invertase; SOC, Organic carbon; UA, Urease.

The Mantel test is widely used in the field of soil ecology. Using the Mantel test, we evaluated the degree of association between bacterial communities and environmental variables (Figure 9). The upper triangle demonstrates the correlation between soil environmental factors based on Spearman's analysis. The lower triangle demonstrates the association between bacterial species composition, species number and diversity, and soil environmental factors. Specifically, soil bacterial community composition in shrub patches and herb patches was susceptible to soil nutrients, pH, soil enzyme activity, and soil particle distribution. In addition, soil nutrients and soil enzyme activities regulated soil bacterial α‐diversity in shrub patches due to higher soil nutrients and enzyme activities. The RDA analysis revealed 87% and 95.6%, respectively, explained by RDA1 and RDA2 for shrub patches and herb patches. Soil enzyme activity and soil nutrients were significantly correlated for the relative abundance of more bacterial phylum. However, there was a significant correlation between *Myxococcota* and soil particle distribution under shrub plots, and this correlation was also present for *Actinobacteriota*, *Firmicutes*, and *Acidobacteriota* under herb plots (Figure 10). In summary, there are differences in the factors regulating soil bacterial community composition and diversity among plant patch types.

## 4 DISCUSSION

**FIGURE 11:**
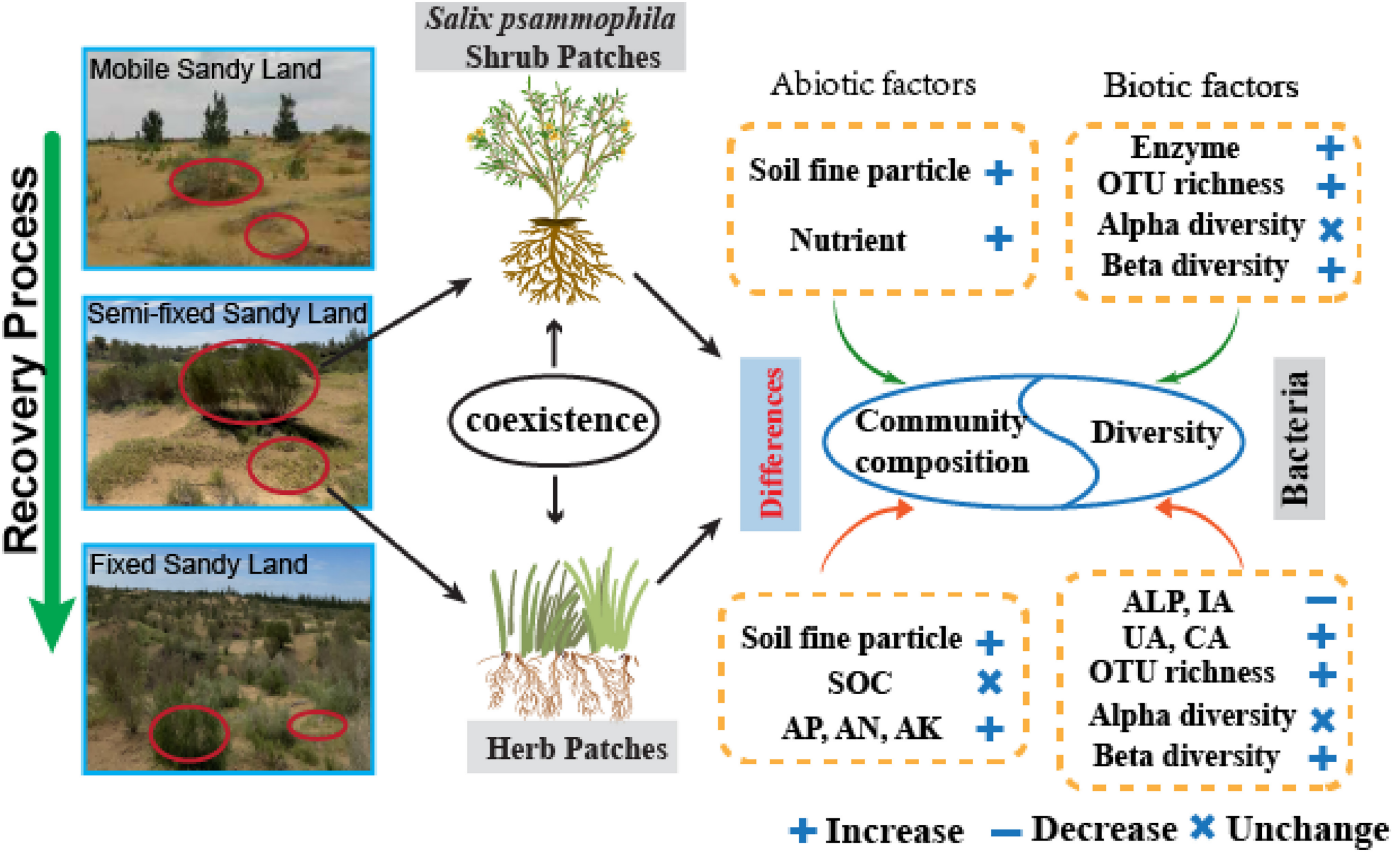
The conceptual framework for characterizing changes in soil bacterial communities of shrub and herb patches under recovery.

Frequent wind erosion is a significant driver of biodiversity loss in the desert (Lupwayi et al., 2023; Sankey et al., 2012). Therefore, restoring the soil environment in desert ecosystems is paramount, as it is an indispensable material basis and an important guarantee for the survival and development of regional vegetation and determines the direction of the desert development. Existing studies have demonstrated that soil bacterial communities are involved in most biogeochemical cycling processes (Li, Jia, et al., 2022; Zhang, Li, et al., 2022; Zhao et al., 2019). In our study, soil clay particles, silt particles, and soil nutrients gradually increased in the recovery stages from mobile to fixed (Figure 11). Nowadays, there is sufficient evidence that recovery stages drive differences in soil particle refinement and soil nutrient content between shrubs and herbs (e.g., differences in aboveground biomass, root secretions, differences in vegetation cover, and differences in the mechanisms of surface soil particle interception) (Gonzales et al., 2018; Madalcho, 2016; Wang et al., 2022; Wen et al., 2022; Zhao et al., 2019). In addition, extracellular enzymes are secreted by soil bacteria, and enzymes degrade macromolecular organic matter, facilitating microbial nutrient management and energy flow in soil ecosystems (Yang et al., 2020). In our study, the UA, ALP, IA, and CA of shrub patches and herb patches with different recovery stages showed inconsistent behavior with soil nutrient distribution. The UA, ALP, IA, and CA of the shrubs increased significantly with recovery stage, which is in agreement with Zhang et al. (2015) report. However, the ALP and IA in the herb patches decreased significantly with recovery stage. Previous studies have shown that the IA and ALP are critical to C and P cycling and that higher enzyme activity accelerates soil carbon and phosphorus turnover (Wang et al., 2020). In our study, there were no significant differences in the SOC and AP in different recovery stages in herb patches, and annual herb plants may not absorb and consume a large amount of organic carbon and available phosphorus, which may lead to small changes in the IA and ALP.

Based on existing conceptual models, biotic and abiotic factors (e.g., soil type, nutrient resource, and soil microbial decomposition) strongly influence bacterial communities (Yang, Li, Wang, Cheng, et al., 2021; Yang, Li, Wang, Dou, et al., 2021). Our results confirm that the Shannon index, which reflects the α‐diversity of soil bacteria, is insensitive to the degree of recovery. Although previous studies have demonstrated that different recovery stages significantly affect the Shannon index of soil bacteria (Gao et al., 2021; Yu et al., 2022; Zhu et al., 2022), in this study there was no significant difference in the Shannon index of soil bacteria in the recovery stage. We believe that the weak response of the Shannon index to restoration may be due to the influence of the soil bacterial Shannon index on plant species abundance during restoration in the desert. In this study, vegetation community characteristics did not develop from simple to complex. From mobile to fixed, phylogenetic diversity of soil bacterial communities increases progressively chronologically, and vegetation patch types lead to distinct patterns of diversity (Poosakkannu et al., 2017; Wang et al., 2015). In addition, due to the unique climatic conditions (i.e., high temperature and drought) of desert ecosystems, the responses of bacterial communities to the environment directly affect ecosystem processes (Tóth et al., 2017). We also found that the ACE index, which responds to the α‐diversity of soil bacteria, is more susceptible to the recovery stage than the Shannon index. However, this is inconsistent with the results of studying desert restoration in northern China (Yu et al., 2022). We suggest that a possible reason for this discrepancy is that plant species characteristics influence the Shannon index of the soil bacterial community (Zhang, Xu, & Xu, 2022). The life forms of *Salix psammophila* and *Artemisia ordosica* are shrubs and semi‐shrubs, respectively, with *Salix psammophila* being larger than *Artemisia ordosica* due to its plant canopy and branching structure (Xiao et al., 2005). Thus, *Salix psammophila* tends to form “fertile islands” for bacterial community activity at different recovery stages. There was no significant difference between the ACE index and Shannon index in the shrub and herbs during the mobile and semi‐fixed (*p* > .05). However, there was a significant difference between the ACE index and Shannon index in the shrub and herbs during the fixed (*p* < .05). In this study, soil physical, chemical, and enzymatic activities were significantly or very significantly higher in shrubs than in herbs during the fixed, and these factors also contributed to the diversity of bacterial communities in shrubs. Aboveground biomass was significantly higher in the shrub patches than in the herb patches, significantly increasing water retention in the shallow soil (Segoli et al., 2008). In addition, shrubs alter the distribution of precipitation resources, with more soil moisture and rainfall distributed under the shrub canopy (Xie et al., 2022). Therefore, this may also lead to more bacterial community diversity in shrubs than in herb patches. The community composition of soil bacteria differed highly significantly across recovery stages (i.e., mobile, semi‐fixed, and fixed) and different vegetation patch types (i.e., shrubs and herbs), as well as interactions (*p* < .001). This agrees with the conclusion reported by Yu et al. (2022). Mantel's analysis emphasized that different plant patch types have different regulatory factors on soil bacterial community composition and diversity. There is currently global agreement that pH significantly affects bacterial community diversity (Jeanbille et al., 2016). Soil enzyme activities are involved in significant processes related to the uptake of C, N, and P by organic matter and link microbial nutrient uptake strategies to resource availability (Stock et al., 2019). Once enzymes are released from microbes, these enzymes will regulate changes in soil bacterial community composition through biological signaling pathways (Tian et al., 2020; Yang, Li, Wang, Dou, et al., 2021). Thus, the ALP and UA significantly influence soil bacterial community diversity.

In this study, vegetation restoration in the desert significantly affected soil bacterial community composition changes. Differences in soil environments due to restoration stages and vegetation types have resulted in inconsistent trends in soil bacterial community composition. The dominant phyla of bacterial communities at different recovery stages and under different vegetation patch types were *Actinobacteriota*, *Proteobacteria*, *Bacteroidota*, and *Gemmatimonadota*. The relative abundance of *Actinobacteriota* and *Bacteroidota* is higher in the early stages of the restoration and prefers nutrient‐poor environments (Poosakkannu et al., 2017; Yu et al., 2022). The relative abundance of *Actinobacteria* and *Bacteroidota* decreased with decreasing soil pH and increasing soil fertility in shrubs and herbs, which is consistent with the findings of Llado et al. (2018). Previous studies have demonstrated a decrease in the relative abundance of *Actinobacteriota* with SOC accumulation, with the *Actinobacteriota* being more abundant in shrub patches than herb patches. In addition, we found that the *Bacteroidota* was more abundant in herb patches than shrub patches. Relevant evidence also demonstrates that the presence of *Bacteroidota* is closely related to unstable organic carbon (Yu et al., 2022). Less litter under shrubs is more readily decomposed. As a result, smaller amounts of unstable organic carbon groups usually remain in the soil (González‐Rosado et al., 2020). Therefore, the significantly higher SOC accumulation in the shrub patches than in the herb patches may be related to the lower content of unstable groups in the soil (Xiao et al., 2023). The relative abundance of the *Proteobacteria* increased significantly from mobile to fixed. Previous studies have demonstrated the r‐strategy by which *Proteobacteria* are thought to prioritize the use of readily available carbon resources, influencing the overall diversity of soil bacteria by accelerating the accumulation of readily available carbon sources (Liu, Liu, et al., 2021). In shrub and herb patches in this study, soil enzyme activities could broadly reflect the balance of bacterial community structure, which is consistent with the findings reported by Yang et al. (2020). In addition, soil particle distribution determines total soil porosity, which affects soil respiration, and soil bacterial communities have a co‐controlling effect on soil respiration (Yang et al., 2019; Zhao et al., 2018). Overall, the soil bacterial community shifted from a poor‐nutrient environment to a rich‐nutrient environment with a recovery stage in both shrub patches and herb patches, and this shift was also closely related to the availability of soil resources (Figure 11).

## 5 CONCLUSION

In vegetation restoration, transitioning from mobile sandy land to semi‐fixed sandy land and finally to fixed sandy land is an essential process in desert restoration. The coexistence of shrub patches and herb patches in this process is a common phenomenon. In this paper, we investigated the soil physicochemical properties, enzyme activities, and soil bacterial communities of shrub patches (dominated by *Salix psammophila*) and herb patches undergoing different stages of restoration (i.e., mobile sandy land, semi‐fixed sandy land, and fixed sandy land) in the deserts of northern China. In our study, mobile sandy land to fixed sandy land promoted soil bacterial community diversity in shrub patches and herb patches, and with restoration bacterial community diversity was significantly higher in herb patches than in shrub patches. Different restoration stages, vegetation‐type patches and interactions significantly influenced soil bacterial community composition. In detail, the dominant bacteria (phyla) in the soil bacterial community under shrub patches and herbaceous patches in different stages of restoration were *Actinobacteria*, *Proteobacteria*, and *Bacteroidota*. The relative abundance of *Bacteroidota* and *Actinobacteriota* decreased gradually from mobile to fixed sands, while the relative abundance of *Proteobacteria* increased significantly. In addition, there was a facilitating effect on the bacterial community composition (genus) of different vegetation patch types during this restoration phase, with herb patches having a more substantial facilitating effect than shrub patches. In summary, our results demonstrate that soil bacterial communities under shrub patches and herb patches show a strong response to the recovery stage. During the restoration process, the increase in soil nutrient content, soil fine particles, and soil enzyme activity under shrub patches and herb patches promoted the development of soil bacterial community structure. This study complements the characterization of the dynamics and drivers of soil bacterial communities restored under shrub patches and herbaceous patches in sandy areas.

## AUTHOR CONTRIBUTIONS

Writing—original draft Haonian Li; investigation Haonian Li, Feiyan Zhao, and Zhongju Meng; methodology, Haonian Li; formal analysis, Ruibing Meng; data curation, Zhongju Meng and Xiaomen Ren; writing—review and editing, Zhongju Meng; supervision, Zhongju Meng and Yumei Liang. All authors have read and agreed to the published version of the manuscript.

## FUNDING INFORMATION

This research was supported by the Basic Research Funds for Universities‐Innovation Team Building – Desert Ecosystem Protection and Restoration Innovation Team, NO. BR22‐13‐03. Study on the movement and morphological characteristics of sand dunes in desert areas based on consumer drones, NO. YSS202307. Technical Challenge Overcoming Project of Inner Mongolia Autonomous Region, NO. 2021GG0073. Inner Mongolia Autonomous Region Science and Technology Program, NO. 2022YFHH0076. And Study on the movement and morphological characteristics of sand dunes in desert areas based on consumer drones, NO. NJZZ20038.

## CONFLICT OF INTEREST STATEMENT

The authors declare no competing interests.

## Data Availability

All data that support this study have been uploaded to the Dryad repository at: https://datadryad.org/stash/share/5TC5NnuLAdj9fT5lbk5cpLss5N2ozOee_whu_kLdTcY.
